# Enhancing infection prevention and control through hand hygiene compliance in six Ugandan hospitals using quality improvement approaches

**DOI:** 10.3389/fpubh.2024.1465439

**Published:** 2024-10-22

**Authors:** Hassan Kasujja, J. P. Waswa, Reuben Kiggundu, Marion Murungi, Grace Kwikiriza, Rony Bahatungire, Henry Kajumbula, Fozo Alombah, Mohan P. Joshi, Niranjan Konduri

**Affiliations:** ^1^USAID Medicines, Technologies, and Pharmaceutical Services (MTaPS) Program, Management Sciences for Health, Kampala, Uganda; ^2^Department of Clinical Services, Ministry of Health, Kampala, Uganda; ^3^Department of Microbiology, College of Health Sciences, Makerere University, Kampala, Uganda; ^4^National Antimicrobial Resistance Sub-Committee, One Health Platform, Kampala, Uganda; ^5^USAID Medicines, Technologies, and Pharmaceutical Services (MTaPS) Program, Management Sciences for Health, Arlington, VA, United States

**Keywords:** infection prevention and control, hand hygiene, healthcare-associated infections, multimodal strategies, continuous quality improvement, hand hygiene compliance, Uganda

## Abstract

**Introduction:**

Hand hygiene (HH) plays a crucial role in mitigating healthcare-associated infections. Improving HH compliance in healthcare facilities in resource-limited settings is urgently needed.

**Methods:**

We implemented the World Health Organization (WHO) HH improvement strategy using a continuous quality improvement (CQI) approach targeting improvement in HH compliance by healthcare workers (HCWs). An intervention was implemented in six hospitals using a longitudinal study design between May 2019 and April 2023. We set up and monitored infection prevention and control (IPC) and HH programs using WHO’s infection prevention and control assessment framework at the facility level (IPCAF) and hand hygiene self-assessment framework (HHSAF) tools. We implemented HH interventions using CQI techniques while targeting HCW HH knowledge and compliance with the WHO’s Five Moments of HH.

**Results and discussion:**

By the end of the intervention, IPC and HH capacity improved in all six hospitals, from a median score of 547.0 and 252.5 on IPCAF and HHSAF tools at baseline to an advanced score of 635.0 and 350.0 at endline assessment, respectively. Similarly, HCWs’ HH knowledge improved in all hospitals, from a mean score of 45.0% at baseline to 76.0% at endline assessment, most notably among nurses. HH compliance, as assessed using WHO’s HH observation tool, at least doubled in all hospitals, rising from 19.9% to 53.8%, with before touching a patient registering the highest (22-fold) improvement. On linear regression analysis, no significant association was observed between HH compliance and IPCAF *b* = -0.0004 (95% CI -0.093, 0.93) *p* = 0.990, HHSAF *b* = 0.009 (95% CI -.0127, 0.145) *p* = 0.842 and HCW knowledge on HH/IPC *b* = -0.165 (95% CI 0.815, 0.485) *p* = 0.519. This is the first documented comprehensive utilization of CQI approaches to implement HH as an entry point for the development of hospital IPC programs, and evaluation of WHO tools and approaches for IPC and HH improvement in Uganda.

**Conclusion:**

Implementation of the WHO HH improvement strategy using a CQI approach can lead to remarkable improvement in HH capacity, and HCW compliance and knowledge in hospitals within resource-limited settings.

## 1 Introduction

Inadequate implementation of infection prevention and control (IPC) programs in healthcare facilities is a major driver of antimicrobial resistance (AMR) and healthcare-associated infections (HAIs) (1). HAIs are associated with poor treatment outcomes and are a significant economic and disease burden to patients, healthcare workers (HCWs), and healthcare givers, especially in low-and middle-income countries (LMICs), with a prevalence of up to 15.5% reported in some settings (2). According to a comprehensive systematic review and meta-analysis of point prevalence studies of HAIs among hospitalized patients in Africa, Uganda stood out with the highest prevalence of 28% (3). A previous study conducted in a district hospital in Uganda reported an even higher prevalence of 34% (4). Worse, the majority of these infections are from multi-drug resistant microbes, making HAIs a major driver of AMR (5).

The high prevalence of HAIs and rising levels of AMR in LMICs can be largely attributed to the inadequate implementation of IPC measures (6). HAIs can spread through direct contact among patients or between patients and HCWs as well as from hospital surfaces, environments, and medical equipment. The hands of HCWs are a major link to this contact hence a major driver for the spread of HAIs and AMR (7). Hand hygiene (HH) has been documented as an effective measure for reducing the transmission of pathogenic microorganisms and lowering the incidence of HAIs in healthcare settings (8). Despite the benefits provided by it, the practice of HH remains low in LMICs, including Uganda (9–11), due in part to a lack of knowledge about HH, poor attitude, and limited supplies of HH materials such as soap and running water in some settings. Prioritizing hospital IPC programs, with a strong emphasis on HH implementation, is therefore crucial as a central strategy for managing and preventing HAIs. Implementing IPC and HH using a multimodal approach that combines strategies such as system change, education and training, evaluation and feedback, reminders in the workplace, and enhanced safety climate and culture is effective in improving HH practices (12, 13).

Systematic capacity building with continuous quality improvement (CQI) initiatives are effective approaches to improving HH practices and reducing the incidence of HAIs in various settings (14, 15). CQI techniques such as the Plan-Do-Study-Act (PDSA) cycle and SWOT (strengths, weaknesses, opportunities, and threats) analysis have been utilized to successfully improve IPC, HH and antimicrobial use in resource-limited settings in sub-Saharan Africa (16–18). However, the impact of such CQI-driven approaches on IPC and HH practices in Ugandan hospitals remains underexplored. The objective of the study was to develop and evaluate a CQI-oriented program based on the WHO approach to improve IPC and HH compliance in selected hospitals. In this paper, we describe the systematic approach that we used to implement the CQI-based IPC program in six hospitals in Uganda, which led to significant improvements in IPC capacity and HH practices.

## 2 Materials and methods

### 2.1 Hospital participation

The United States Agency for International Development’s Medicines, Technologies, and Pharmaceutical Services Program (hereafter referred to as the project) supported 13 hospitals in Uganda—six public government-owned regional referral hospitals and seven private not-for-profit (PNFP) hospitals—in implementing IPC activities. The public hospitals were chosen purposively based on their ongoing involvement in related IPC programs, and the private hospitals were chosen based on geographic location to cover the four main regions of the country.

Out of these 13 hospitals, six were selected, based on availability of resources and funding, to participate in the IPC/HH CQI program and receive targeted technical assistance to advance their IPC capacity, with close attention to improving HH. Selection of the six hospitals was based on the commitment of the facilities’ management to improving IPC, willingness to participate in the program, and approval by the Uganda Ministry of Health (MOH). Table 1 shows the characteristics of the six participating hospitals. Five were PNFP hospitals, while one was a public hospital. Bed capacities varied from 100 to 482, and staff numbers ranged from 162 to 705. The large size and capacity of the hospitals are further illustrated by additional characteristics, including annual admissions, surgical operations, and outpatient visits.

**Table 1:** Characteristics of hospitals that participated in the IPC/HH CQI program.

	Ownership	Hospital bed capacity	No. of staff	Admissions	Surgeries	OPD visits
			2020/21	2020/21	2020/21	2020/21
Naggalama	PNFP	100	162	3,814	3,044	58,074
Kagando	PNFP	231	284	8,050	1,930	10,860
Kiwoko	PNFP	204	300	7,599	1,581	27,169
Kumi	PNFP	330	179	3,512	1,040	19,105
Lacor	PNFP	482	705	18,456	7,811	146,674
Gulu RRH	Public	347	273	19,211	3,244	152,011

OPD, Outpatient department; PNFP, Private-not-for-profit; RRH, Regional referral hospital.

The project utilized a longitudinal design and conducted the study in a two-phase approach to implement activities from May 2019 to April 2023.

### 2.2 Ethics statement

The Uganda MOH granted permission to the project for long-term, multi-year technical assistance in multisectoral coordination on AMR, IPC, and antimicrobial stewardship. In line with Uganda’s National Action Plan on AMR, this includes permission for antibiotic use and IPC studies, and designing interventions suitable in the participating hospital settings. The conduct of this study and intervention is part of the project’s routine technical assistance in line with the Uganda MOH’s priorities. The senior administration of all participating hospital sites provided their respective approval and clearance. There was no direct patient contact. All data were anonymized including that of HCWs. The study was conducted according to the guidelines of the Declaration of Helsinki.

### 2.3 Phase 1: initiating the hospital IPC programs

**Figure 1:**
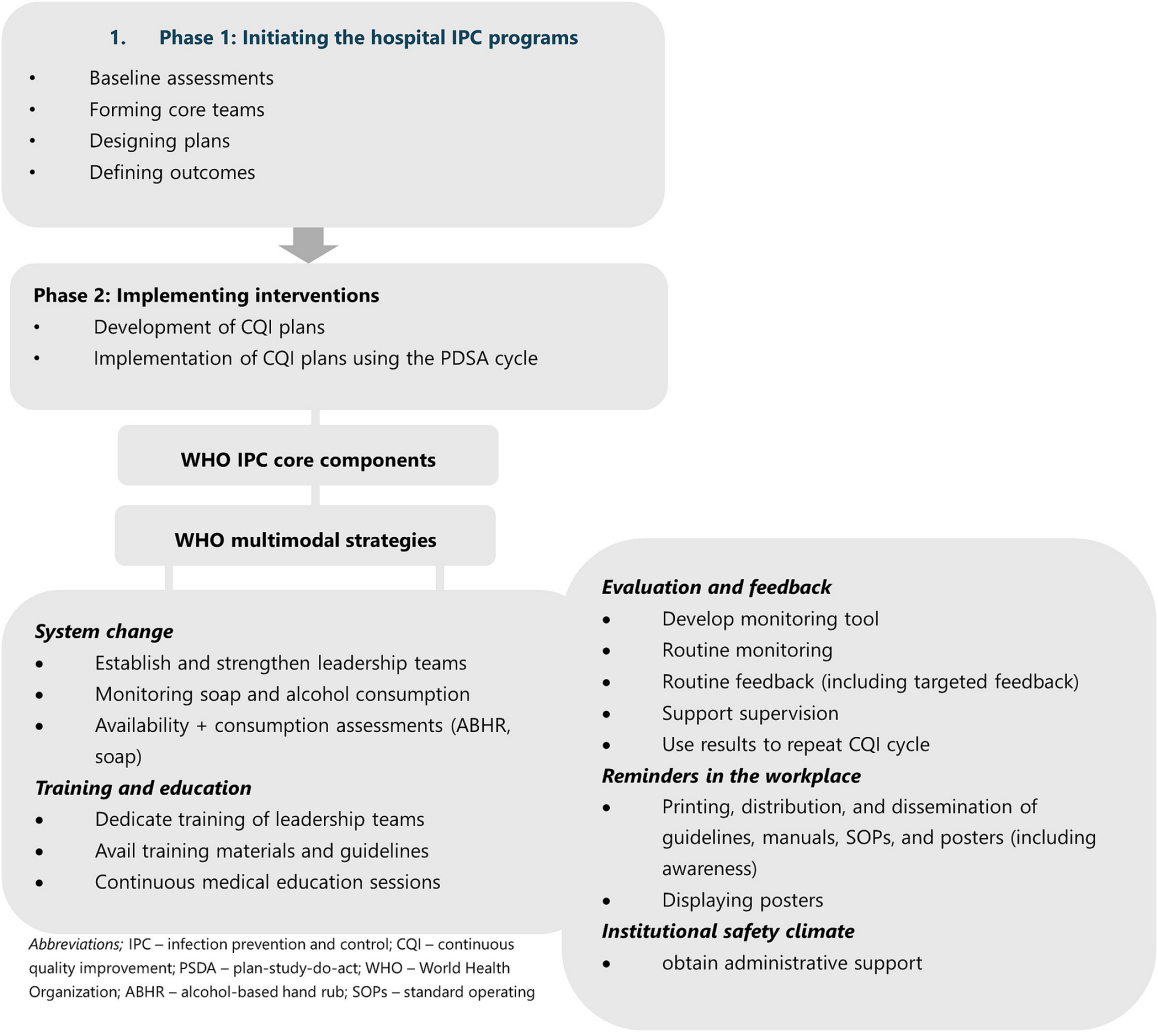
Flow chart of intervention phases and key activities.

Figure 1 summarizes the intervention phases and key activities carried out. During Phase 1, customized roadmaps for the implementation of IPC and HH programs were developed for individual hospitals, guided by the WHO IPC core components (19) and the WHO multimodal strategy for improving hand hygiene (20). The phase encompassed various key steps, including conducting baseline assessments on IPC and HH, assembling and establishing core teams, strategizing implementation plans, defining desired outcomes, preparing hospitals for IPC and HH interventions, and building a compelling business case for IPC and HH through the demonstration of potential outcomes and impacts on patient safety and AMR containment. This phase was implemented from May 13, 2019 to May 29, 2020.

#### 2.3.1 Setting up hospital IPC program

Following the baseline assessments, the project focused efforts in helping establish governance mechanisms for the hospital IPC program. The project provided technical assistance to develop IPC and HH work plans that focused on defining structures, systems, and roles within which the hospital IPC and HH teams would operate. The activities included obtaining hospital management buy-in and ownership of IPC and HH activities, revitalizing IPC committees and IPC teams, and establishing HH teams that worked under the IPC committees. The members of these committees and teams were selected and recommended by the IPC focal persons in alignment with national (21) and WHO guidance (19, 20) and were formally appointed by the hospital administrations. Priority for committee and team membership was given to departmental/unit heads and motivated and interested individuals (champions). Led by the hospital IPC focal persons, IPC teams each comprised at least four HCWs tasked with coordinating the implementation of IPC actions and technical decisions of the IPC committee. Similarly, HH teams, led by the focal person for HH, each comprised at least two HCWs responsible for the implementation of HH activities in the hospital. As technical arms of the IPC committee, these two teams reported their progress and recommendations to the IPC committee during its regular plenary meetings. A system was established for holding regular committee meetings, documenting meeting proceedings, and taking actions. Hospital-based continuous medical education and continuous professional development initiatives were set up. The implementation of the IPC core components and HH multimodal strategies was done using the PDSA cycle (22).

### 2.4 Phase 2: implementing interventions to improve HH structures and practices

This phase was implemented between June 8, 2020 and April 24, 2023 and focused on improving HH structures and practices while supporting HH and IPC teams.

#### 2.4.1 Prioritizing interventions and developing the CQI plan

As an entry point to implementing hospital IPC programs, we prioritized programmatic implementation of HH interventions to improve systems, structures, and practices (20). Each hospital undertook training on the CQI plan development process for HH. The training was guided by findings from the baseline surveys and included practical aspects such as identifying stakeholders for hospital HH implementation; assessing resource needs; assessing the feasibility of various HH interventions for implementation at the health facility (ranking interventions); making HH interventions specific, i.e., choosing certain interventions for prioritized actions; conducting SWOT analyses for hospital HH programs; identifying barriers and mitigation plans for hand hygiene programs; and developing the CQI plan. While choosing interventions for the IPC and HH program the project prioritized the strategies listed in the as the WHO IPCAF core components and the HH multimodal strategies. The project, in collaboration with the MOH, provided technical assistance to the hospital IPC and HH teams to develop a shorter customized tool, based on the WHO infection prevention and control assessment framework at the facility level (IPCAF) (23) and hand hygiene self-assessment framework (HHSAF) (24) tools, that was used for monitoring. The customized tool facilitated the monitoring of interventions and guided capacity building, mentorship, and supportive supervision conducted by the project and the MOH. Additionally, the project, in collaboration with the MOH, provided technical assistance to the hospitals, including routine mentorships and supervisions; printing and distributing standardized WHO information, education, and communication materials such as guidelines, posters, and other workplace reminders; and printing and distributing MOH guidelines and manuals.

#### 2.4.2 Routine mentorship and supervision

Monthly mentorship and supervision visits were conducted by the project, in collaboration with the MOH. The mentorship and capacity-building activities included continuous medical education sessions, onsite and offsite training for HCWs, benchmark learning activities, and instant feedback for and meetings with the IPC/HH teams and clinicians (Table 2). The training was offered by the project’s technical personnel and members of the IPC and HH team. The latter were supported by the project’s technical personnel to design and deliver the training material and contents, respectively, focusing on the need for HCWs to adhere to IPC and HH guidelines. This training was delivered to all HCWs in the facility including clinicians, nurses, and laboratory staff, from all units of the hospitals.

**Table 2:** Capacity-building interventions conducted by the project technical team.

	Mentorship visits	CMEs	Trainings	Total HCWs trained		
				Male	Female	Total
IY 2020	12	8	15	211	254	465
IY 2021	58	23	28	504	608	1,112
IY 2022	14	15	16	216	286	502
IY 2023	5	5	5	46	56	102
Total	89	51	64	977 (44.8%)	1,204 (55.2%)	2,181

IY, Intervention year (October to September); CME, Continuous medical education; HCW, Healthcare worker.

### 2.5 Data collection

#### 2.5.1 Assessment tools

We applied the WHO IPCAF (23) to assess the IPC core components, HHSAF (24) to assess the capacity of the hospitals to implement HH multimodal strategies, the WHO HH knowledge questionnaire for healthcare workers (25) to assess knowledge of HCWs on HH, and the HH observation tool to assess compliance with the Five Moments of HH (26). The IPCAF and HHSAF tools have been validated and proven reliable for use, while the HH knowledge questionnaire and observation tools have been used in various settings to evaluate HH knowledge and compliance among HCWs, respectively (27–30).

The IPCAF framework is a tool developed by WHO to support the implementation of the eight core components of hospital IPC programs: *IPC program*; *IPC guidelines*; *IPC training and education*; *HAI surveillance*; *multimodal strategies for implementation of IPC interventions*; *monitoring/audit of IPC practices and feedback*; *workload, staffing, and bed occupancy*; and *built environment, materials, and equipment* for IPC at the facility level. Designed primarily for self-assessment purposes, the tool comprises closed-formatted questions with 81 indicators accompanied by a scoring system. Each core component allows for a maximum score of 100 points; the resulting possible maximum tool score of 800 points facilitates a comprehensive evaluation process. The final IPCAF score is determined by summing the scores of the eight core components and then assigning an IPCAF level to the evaluated healthcare facility based on that overall score: inadequate (0–200 points), basic (201–400 points), intermediate (401–600 points), and advanced (601–800 points) (23).

The WHO HHSAF framework supports the systematic assessment of HH promotion and practices in healthcare facilities (HCFs). The self-assessment tool comprises five components—which reflect the five elements of the WHO multimodal HH improvement strategy: *system change*; *training and education*; *evaluation and feedback*; *reminders in the workplace*; and *institutional safety climate for HH* (20)—and 27 indicators. Like the IPCAF tool, each component of the HHSAF tool permits a maximum score of 100 points, resulting in a potential maximum overall score of 500 points. A total score is computed after the assessment to assign the HCF a HHSAF level of inadequate (score: 0–125), basic (score: 126–250), intermediate (score: 251–375), or advanced (score: 376–500) (24).

The WHO HH knowledge questionnaire for HCWs is one of the WHO dedicated tools for monitoring and evaluation of HH interventions in HCFs (31). The tool collects HCW knowledge and information on various aspects of HH: training on HH; use of alcohol-based handrub (ABHR) for HH; handwashing; infection cycle in HCF settings; HH methods and actions in healthcare delivery; and risks for pathogenic colonization of hands. The closed-format questionnaire does not include a scoring system. Consequently, the project, in collaboration with hospital IPC and HH teams, developed a scoring system that assigns one point for each technical question assessing knowledge. For each correct response, one point was awarded to the HCW. A percentage score was then calculated for each HCW assessed based on the number of correct responses, and the mean score for the HCF was determined by computing the mean of all HCWs’ scores assessed during a specified assessment period (25).

Considered a gold standard, the WHO observation tool, extracted from the WHO HH technical reference manual, is used by HCWs, trainers, and observers of HH practices all over the world to monitor the effectiveness of HH interventions on HCWs’ HH compliance (24, 25). Using direct observation techniques, the tool assesses the compliance of HCW with the five moments (indications) of HH: before touching a patient; before clean or aseptic procedure; after body fluid exposure risk; after touching a patient; and after touching patient surroundings. Compliance with proper HH practices during these five moments is intended to mitigate the risk of microbial transmission between HCWs, patients, and the environment during healthcare interactions. The tool tracks the number of HH actions undertaken by an HCW against that HCW’s opportunities for HH during the observation period to determine the level of compliance (as a percentage proportion of HH actions against HH opportunities). The HH actions can be hand washing (with soap and water) or handrub (with ABHR), with or without gloves. The level of compliance can be estimated as mean compliance by cadre, ward, or HCF.

#### 2.5.2 Data collection procedures

Following formal approval for these interventions in line with Uganda MOH priorities, we again obtained verbal consent from the senior hospital management and HCWs for the implementation of the tools. The project trained the facility IPC and HH teams and supported them in collecting essential data, including baseline, monitoring, and evaluation metrics. As a quality control measure, the project team randomly selected indicators that were validated for accuracy by discussing the scores with the healthcare workers that conducted the assessment. Table 3 shows the data collection methods for the applied tools. The IPCAF and HHSAF tools were applied at various implementation stages by the hospital IPC focal person or the nursing in-charge.

**Table 3:** Tools and methods used in the assessments and the respective correspondents.

Tool	Method of assessment	Respondents/study population
Infection prevention and control assessment framework (IPCAF)	Interviewing and observation	IPC focal person/nursing in-charges
Hand hygiene self-assessment framework (HHSAF)	Interviewing and observation	IPC focal person/nursing in-charges/hand hygiene focal persons
Hand hygiene knowledge questionnaire for HCWs	Interview	Hospital staff
Hand hygiene observation tool	Direct observation technique	HCWs

The HH knowledge questionnaire was used to assess the HH comprehension of 20 randomly selected HCWs per hospital at various intervals. The 120 HCWs that participated in the knowledge assessments included doctors, nurses (including midwives), laboratory professionals (technicians, assistants, and scientists), pharmacy professionals (pharmacists, technicians, and dispensers), paramedical health professionals (clinical, dental, and orthopedic officers), records officers, and hospital administration and support staff (non-technical). These HCWs represented various hospital wards/sections including surgery, medical, pediatrics, maternity, and outpatient department (including administration and support staff). The HH observation tool was used on all hospital wards during ward rounds, taking 20 min for each round of observation. The observation covered 183 HCWs (baseline) and 202 (endline) and included the same cadres (excluding pharmacy professionals and administrators/support staff) (see Table 4 for details). We obtained verbal consent from the HCWs for administering both the HH knowledge questionnaire and the HH observation tool. Both tools were used for all cadres of HCWs in all the hospital units in the presence of the appointed HH focal person. These assessments were conducted at baseline and at various stages of implementation. In this paper, results from baseline assessments conducted on May 13, 2019 and endline assessments conducted on May 29, 2023 are presented and compared as a measure of implementation outcomes.

**Table 4:** Characteristics of HCWs participating in HH knowledge survey and HH compliance.

	Hand hygiene knowledge						Hand hygiene compliance					
	Baseline (May 2019)		Endline (May 2023)		*p* value*	95% CI of ΔX	Baseline (May 2019)		Endline (May 2023)		*p* value*	95% CI of ΔX
	HCWs (*n* = 120)	Mean (SD)	HCWs (*n* = 120)	Mean (SD)			HCWs (*n* = 183)	Mean (SD)	HCWs (*n* = 202)	Mean (SD)		
Wards/department												
Surgery	13	57.5% (15.8)	11	64.3% (19.9)	0.243	−27.3 to 13.7	34	19.7% (11.8)	29	52.6% (15.2)	<0.001	28.6–37.8
Medical	25	40.0% (10.4)	18	64.0% (19.5)	0.002	20.9–26.8	31	16.2% (7.8)	31	52.4% (15.1)	<0.001	32.6–41.2
OPD	46	45.6% (14.1)	43	72.0% (11.0)	<0.001	23.5–29.6	59	13.5% (9.0)	63	53.7% (13.3)	<0.001	35.6–46.1
Pediatrics	15	45.5% (8.8)	28	65.3% (11.9)	<0.001	15.8–23.9	32	18.7% (9.4)	38	49.6% (15.9)	<0.001	26.0–37.1
Maternity	21	46.2% (11.9)	20	75.0% (11.9)	<0.001	25.5–32.5	27	21.2% (12.7)	41	51.9% (15.4)	<0.001	25.9–37.1
Cadre												
Doctor	11	50.0% (10.7)	16	67.5% (12.4)	0.002	14.9–19.4	30	12.7% (5.5)	31	38.6% (9.8)	<0.001	20.8–32.2
Nurse	63	45.5% (13.1)	71	66.8% (17.0)	<0.001	17.5–25.8	98	19.7% (10.9)	86	56.4% (13.8)	<0.001	32.9–41.5
Laboratory professional	19	53.0% (9.5)	12	75.0% (10.5)	0.017	20.4–19.7	18	9.0% (5.1)	16	45.5% (13.0)	<0.001	33.3–42.7
Other**	17	46.7% (15.0)	12	76.7% (8.2)	0.070	−13.8 to 46.2	37	18.0% (10.7)	69	54.6% (14.1)	<0.001	33.0–41.7
Pharmacy professional	10	30.0% (12.2)	8	62.5% (9.6)	0.162	−49.7 to 15.3						
Age												
<35 years	68	48.5% (13.3)	75	67.8% (14.0)	<0.001	15.6–23.7						
≥35 years	52	42.8% (13.1)	45	70.7% (15.8)	<0.001	27.9–35.7						
Sex												
Male	43	51.8% (12.8)	52	72.4% (12.0)	<0.001	16.8–25.9						
Female	77	41.2% (11.8)	68	65.7% (15.4)	<0.001	19.6–30.7						

HH, Hand hygiene; SD, Standard deviation; CI, Confidence interval; ΔX, Difference between the means; OPD, Outpatient department; HCW, Health care worker. *Statistical significance evaluated at *p* = 0.05 for differences between means using unpaired student *t*-test. **Other includes paramedical healthcare workers, records officers, and administration and support staff.

### 2.6 Data analysis

Descriptive statistics such as mean ± standard deviation or median (interquartile range) were used to summarize IPC and HH capacities, HCW knowledge on HH, and the level of HCW compliance with WHO’s Five Moments of HH. Comparison was conducted between baseline and endline points to measure intervention outcomes. Student’s t-tests were conducted where appropriate to compare changes in means between different groups. Simple linear regression analysis was conducted to examine the relationships between independent variables (IPCAF and HHSAF) and the dependent variables (HCW knowledge of HH and compliance to HH) separately due to small sample sizes, highly correlated variables, and limited control of study participation. The regression analysis sought to determine whether enhancing hospital capacity, as measured by IPCAF and HHSAF tools, had a quantifiable association with individual HCWs’ HH knowledge and compliance scores. Significance was evaluated at *p* < 0.05 and 95% confidence intervals (CIs) were reported. Analyses were conducted using STATA 14 (StataCorp LLC. 2015) and Microsoft Excel 365 (Microsoft Corporation. 2017).

Our findings have been written following the reporting guidelines from the Enhancing the Quality and Transparency of Health Research Network as per the SQUIRE checklist where applicable (32).

## 3 Results

### 3.1 IPC and HH capacity

All hospitals (6/6) demonstrated improvement on the IPCAF tool, achieving advanced IPC capacity (Table 5). Improvement was observed from the baseline median score of 547.0 (IQR 125.0) to 635.0 (IQR 75.6) at endline assessment. Improvement was also observed on the HHSAF from the baseline median score of 252.5 (IQR 41.2) to 350.0 (IQR 81.2) at endline assessment (Table 5). Three of the six hospitals progressed to achieve advanced scores on the HHSAF tool at endline assessment.

**Table 5:** Baseline and end line scores for IPCAF and HHSAF in six supported hospitals.

Hospital	IPCAF scores (/800)		HHSAF scores (/500)	
	Baseline (May 2019)	Endline (May 2023)	Baseline (May 2019)	Endline (May 2023)
Gulu RRH	602.0	642.5	265.0	350.0
Kumi	395.0	602.5	312.5	370.0
Lacor	590.0	695.5	217.5	410.5
Naggalama	552.5	700.0	217.5	435.0
Kiwoko	342.5	655.0	252.5	435.0
Kagando	497.5	605.5	162.5	345.0
Median (IQR)	547.0 (125.0)	635 (75.6)	252.5 (41.2)	350.0 (81.2)

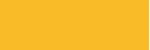
 Basic score: IPCAF (201–400), HHSAF (126–250).

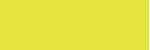
 Intermediate score: IPCAF (401–600), HHSAF (251–375).

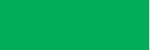
 Advanced score: IPCAF (601–800), HHSAF (376–500).

IPCAF, Infection prevention and control assessment framework; HHSAF, Hand hygiene self-assessment framework; RRH, Regional referral hospital; IQR, Interquartile range.

**Figure 2:**
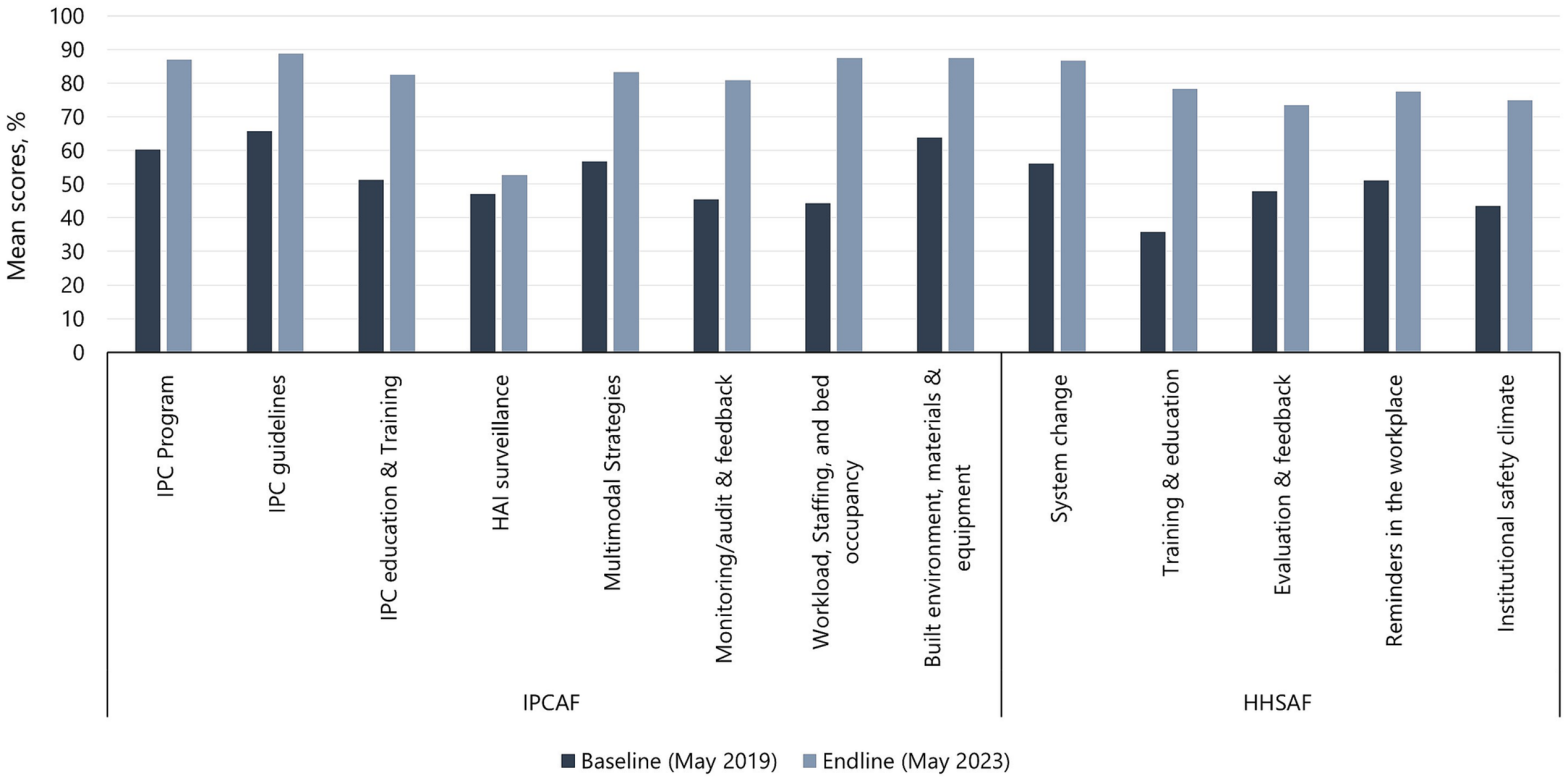
Baseline and endline mean scores for IPCAF core components and HHSAF multimodal strategies.

Improvement from baseline to endline assessments was demonstrated in scores for all the core components (8/8) of the IPCAF tool and all multimodal strategies (5/5) of the HHSAF tool (Figure 2). For IPCAF, the largest percentage improvement in mean scores was observed in the *workload, staffing, and bed occupancy* (97.1%) component, followed by the *monitoring/audit & feedback* (77.6%), *IPC education and training* (60.8%), and *multimodal strategies* (46.6%) components. The *HAI surveillance* (12.1%) component showed the smallest percentage improvement, followed by the *IPC guidelines* (35.1%), *built environment, materials, and equipment* (36.9%), and *IPC program* (44.7%) components. For HHSAF, all components showed at least 50% improvement in mean scores from baseline to endline assessment, with *training and education* having a 2-fold improvement, followed by *institutional safety climate* (72.4%) and *system change* (54.3%). *Reminders in the workplace* (51.4%) and *evaluation and feedback* (53.2%) showed the lowest observed percentage improvement.

### 3.2 HCW knowledge on HH

**Figure 3:**
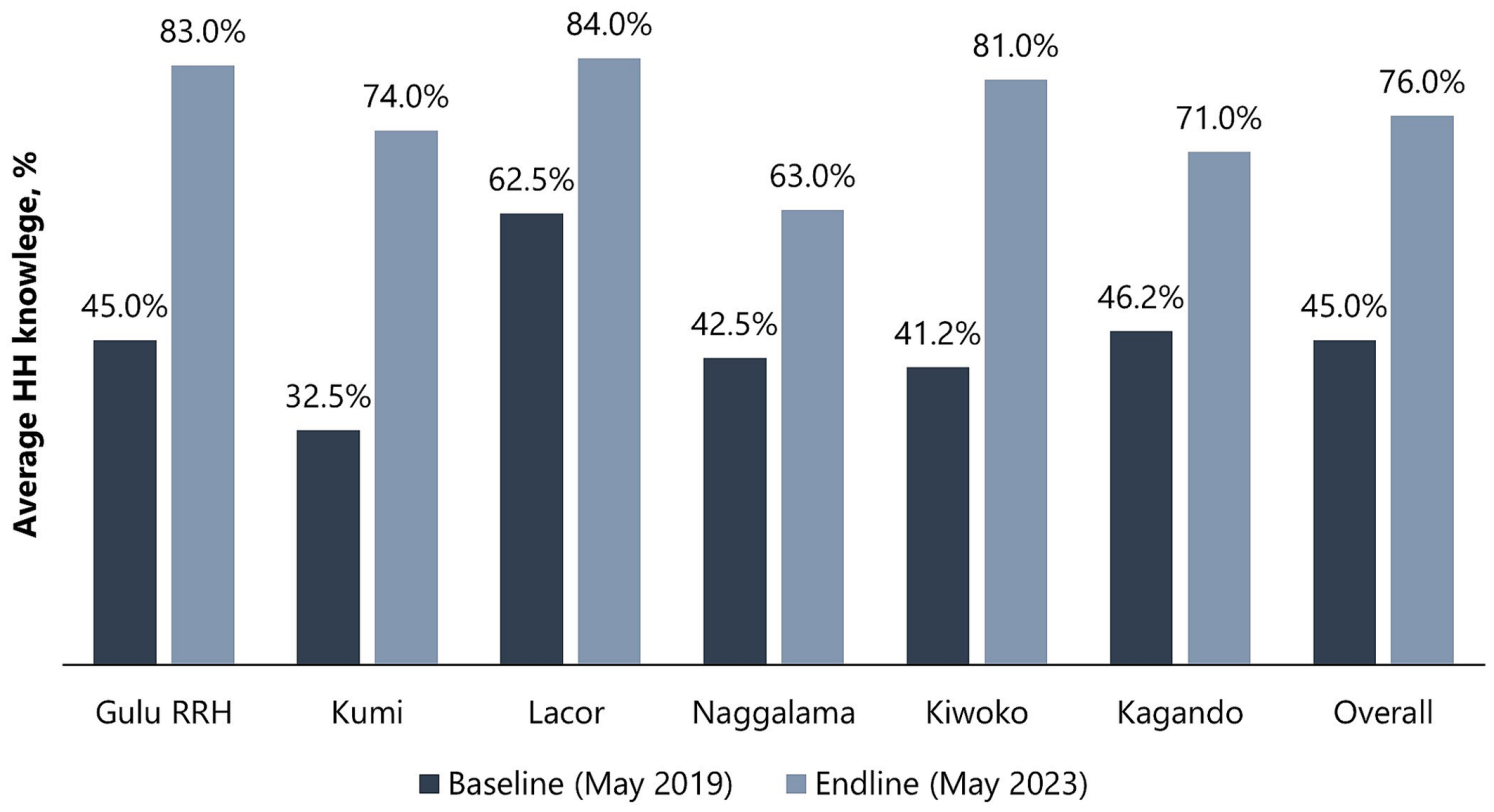
Baseline and endline mean scores for HCW knowledge on HH.

Healthcare workers from all hospitals (6/6) demonstrated improvement in HH knowledge, with an increase in mean scores from 45.0% (SD 9.8) at baseline to 76.0% (SD 8.2) at endline (Figure 3). At baseline, only one hospital (1/6) had a mean score of above 50% (range: 41.2–62.5%), but all hospitals had mean scores of above 50% (range: 63.0–84.0%) at endline, with 3/6 hospitals achieving mean scores of at least 80%—Gulu Regional Referral Hospital (83.0%), Lacor Hospital (84.0%), and Kiwoko Hospital (81.0%). Table 4 shows the characteristics of HCWs assessed for HH knowledge and the differences between baseline and endline results including the *p*-value and 95% confidence interval of the difference between the means [CI (*ΔX*)]. Female HCWs showed 59.5% [*p* < 0.001, 95% CI (*ΔX*) 19.6–30.7] improvement in mean HH knowledge compared to 40% [*p* < 0.001, 95% CI (*ΔX*) 16.8–25.9] improvement among male HCWs. In terms of HCWs’ age, older individuals (>35 years) had a bigger improvement (65.2%) [*p* < 0.001, 95% CI (*ΔX*) 27.9–35.7] in mean HH knowledge compared to younger HCWs (39.8%) [*p* < 0.001, 95% CI (*ΔX*) 15.6–23.7]. Nurses (46.8%) [*p* < 0.001, 95% CI (*ΔX*) 17.5–25.8] had the highest improvement in mean HH knowledge, followed by laboratory professionals (41.5%) [*p* = 0.017, 95% CI (*ΔX*) 20.4–19.7], and doctors (35.0%) [*p* = 0.002, 95% CI (*ΔX*) 14.9–19.4]. Significant improvement in HCW HH knowledge was observed in the outpatient department, pediatrics, maternity, and medical wards. On linear regression analysis, no significant association was observed between knowledge on HH and HHSAF *b* = −0.116, (95% CI –0.247, 0.016) *p* = 0.079, and IPCAF *b* = 0.044 (95% CI –0.084 to 0.172) *p* = 0.399.

### 3.3 HCW HH compliance

Table 4 shows the characteristics of HCWs observed for HH compliance at baseline and endline assessments, with the differences between the mean compliance being significant (*p* < 0.001) for all categories. Table 6 shows baseline and endline scores for HH compliance in the six hospitals. Up to 183 healthcare workers were observed at baseline, generating 2,265 opportunities, and 202 healthcare workers were observed at endline, generating 2,354 opportunities. The majority of HCWs observed during both baseline and endline assessments were from the outpatient department, with wards being relatively uniformly represented. Additionally, nurses constituted the largest group of observed HCWs, followed by pharmacy professionals and doctors, as shown in Table 4.

**Table 6:** Baseline and endline scores for hand hygiene compliance in six supported hospitals.

	Baseline (May 2019)				Endline (May 2023)			
Hospital	No. of HCWs observed	Opportunities	Actions	Compliance	No. of HCWs observed	Opportunities	Actions	Compliance
Gulu RRH	39	348	53	15.2%	42	411	196	47.7%
Kumi	28	402	68	16.9%	34	355	194	54.6%
Lacor	33	399	72	18.0%	38	466	249	53.4%
Naggalama	21	180	26	14.4%	26	174	100	57.5%
Kiwoko	35	557	150	26.9%	37	590	344	58.3%
Kagando	27	379	81	21.4%	25	358	184	51.4%
Total	183	2,265	450	19.9%	202	2,354	1,267	53.8%

RRH, Regional referral hospital; No., Number; HCW, Healthcare worker.

Healthcare worker demonstrated improvement in compliance in all (6/6) hospitals, with observed overall compliance more than doubling from 19.9 to 53.8% (Table 6). All hospitals demonstrated improvement in mean HH compliance, with Naggalama Hospital seeing a four-fold improvement (14.4–57.5%), followed by Kumi Hospital (16.9–64.6%), Lacor Hospital (18.0–53.4%), and Gulu Regional Referral Hospital (15.2–47.7%), which each saw a 3-fold improvement from baseline to endline assessments. The least improvement was observed in Kagando Hospital (21.4–51.4%) and Kiwoko Hospital (26.9–58.3%), with both demonstrating a 2-fold improvement in mean HH compliance among observed HCWs. The mean HH compliance among laboratory professionals increased 5-fold, from 9.0 to 45.5% [*p* < 0.001, 95% CI (*ΔX*) 33.3–42.7]. Additionally, mean compliance among doctors and other HCWs tripled, rising from 12.7% (SD 5.5) to 38.6% (SD 9.8) [*p* < 0.001, 95% CI (*ΔX*) 20.8–32.2] and from 18.0% (SD 10.7) to 54.6% [*p* < 0.001, 95% CI (*ΔX*) 33.0–41.7], respectively. Moreover, mean compliance among nurses nearly tripled, increasing from 19.7% (SD 10.9) to 56.4% (SD 13.8) [*p* < 0.001, 95% CI (*ΔX*) 32.9–41.5]. With respect to hospital wards, the largest improvement in mean HH compliance was observed in the outpatient department, with a 4-fold increase from 13.5% (SD 9.0) to 53.7% (13.3) [*p* < 0.001, 95% CI (*ΔX*) 32.9–41.5]. The performance in the wards is shown in Table 4. On linear regression analysis, no significant association was observed between HH compliance and IPCAF *b* = −0.0004 (95% CI –0.127, 0.145) *p* = 0.990, HHSAF *b* = 0.009 (95% CI –0.127 to 0.145) *b* = 0.842 and HCW knowledge on HH/IPC *b* = −01.6 (95% CI –0.815 to 0.485) *p* = 0.519.

### 3.4 HH compliance by indication

**Figure 4:**
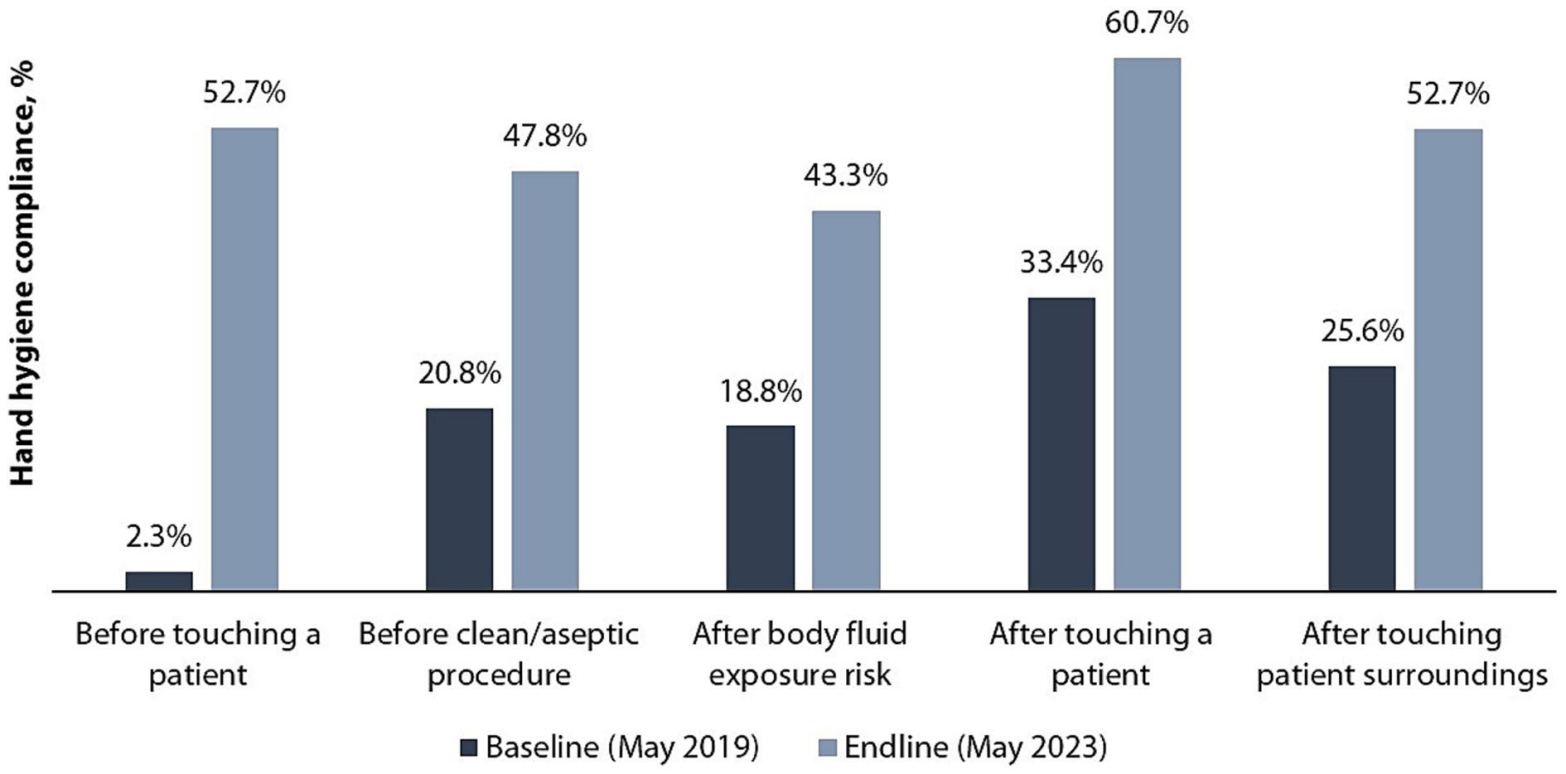
Baseline and endline scores for hand hygiene compliance by indication.

**Figure 5:**
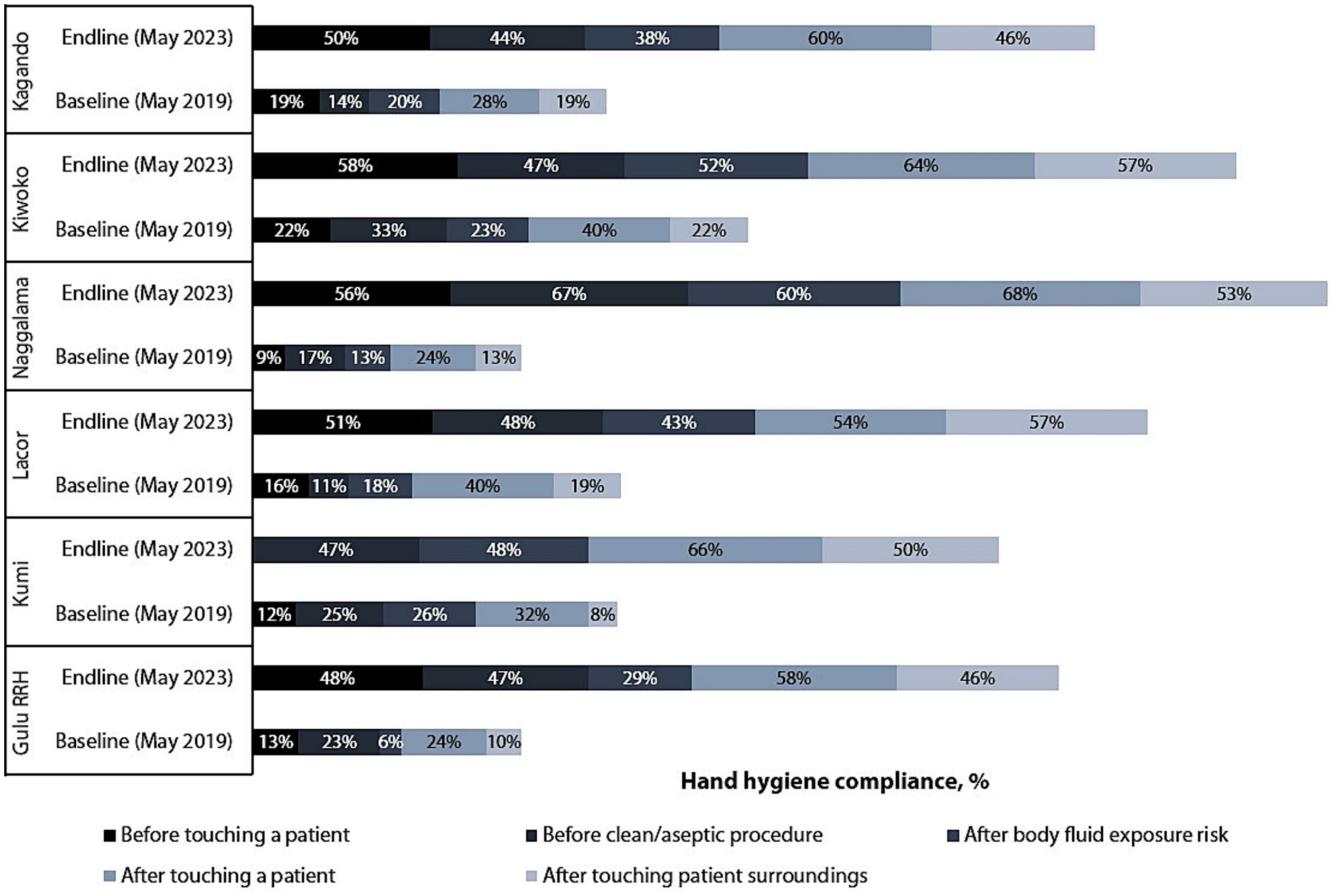
Baseline and endline scores for hand hygiene compliance by indication in six hospitals.

Figure 4 shows improvement in HCW HH compliance in all the five indications (moments) of HH, mostly noted for *before touch a patient indication* (22-fold), with the rest doubling between the baseline and endline assessments. Baseline and endline scores for HCW HH compliance by indication in the six hospitals are shown in Figure 5. Improvement in HH compliance from baseline to endline is observed in all (6/6) hospitals for all HH indications.

## 4 Discussion

To the best of our knowledge, this is the first documented comprehensive utilization of CQI approaches to implement HH as an entry point for the development of hospital IPC programs, and evaluation of WHO tools and approaches for IPC and HH improvement in Uganda. Our work demonstrates the feasibility of applying CQI approaches to advance health facilities capacity of IPC and hand hygiene in a resource-limited setting using the WHO IPCAF and HHSAF tools and to improve HH knowledge and HH compliance.

### 4.1 Impact of CQI interventions on IPC capacity

The observed improvement in scores using the IPCAF tool in HFCs in Uganda has not been previously described. However, IPCAF tool, applied at regular intervals, have been used to assess and improve IPC performance in Sierra Leone (33). Much like the results in Sierra Leone, our results show a demonstrated improvement in all IPC core components. Similarly, follow-up IPCAF assessments at five hospitals in Senegal showed improved scores and capacity level rise in each hospital compared to baseline values, demonstrating the positive effects of the intervening improvement actions (34). The current work provides further evidence of the applicability of this tool in a resource-limited setting. The observed low improvement on the *HAI surveillance* core component is not surprising for a resource-constrained setting like Uganda—the challenges in establishing HAI and AMR surveillance systems in Uganda and other LMICs have been documented (35). Additionally, while the WHO offers practical guidance on establishing HAI surveillance programs in HCFs, it inadvertently lacks detailed instructions on resource identification, a crucial aspect for the successful implementation of such programs (19). The improvement in the *monitoring/audit & feedback component* was due to the program approach of emphasizing the use of data to drive action. The project support toward routine audits and feedback may have contributed to this observed improvement, a finding that has been reported in other settings (36, 37). The most significant change was in the *workload, staffing and bed occupancy* component. This may have been due to the government’s efforts to contain the COVID-19 outbreak in the country, which included increased staffing at HCFs and the availability of hospital equipment, such as additional beds to accommodate the surge of patients. Furthermore, strict policies promoting patient spacing were implemented. These changes occurred during the implementation of our IPC program and their impact on improving IPC capacity is evident.

### 4.2 Impact of CQI interventions on HH capacity and compliance

Improvement was demonstrated in HH capacity on the HHSAF tool in all six supported hospitals. Utilization of the WHO multimodal strategy and the HHSAF tool to improve HH capacity and practices has been described elsewhere (38). As expected, variation in the level of implementation and demonstrated improvement among the hospitals was seen due to hospital-specific differences in the capacity to adopt and implement HH interventions. The program was able to demonstrate improvement in all five multimodal strategies, most notably in the *training and education* component. The program prioritized education and training interventions on HH due to the documented benefits in enhancing HH knowledge and improving HH practices among HCWs and patients (39, 40).

In most of the HCFs, the increase in IPCAF scores positively associated with increase in HHSAF scores. This aligns with findings from similar interventions in other settings (41). Despite achieving advanced capacity on the IPCAF tool, two of the study hospitals (Gulu RRH and Kumi Hospital) maintained the same baseline HH capacity (Intermediate) despite some improvements in their HHSAF scores. This could be attributed to the variability in HCWs’ interest in HH intervention activities, as compared to IPC activities. Our intervention heavily relied on motivated and passionate individuals (champions) to drive actions forward. However, there was significant variation in the availability of these champions to lead IPC or HH initiatives both within and across HCFs. In these two hospitals, IPC champions emerged early, while HH champions were slower to develop. Additionally, the HH champions who had managed to gain momentum were eventually lost due to staffing changes, impacting implementation progress. The critical role of champions in driving quality improvement interventions in resource-limited settings has been documented in similar settings (17, 42).

The observed 2-fold improvement in HH compliance between the baseline (18.8%) and the endline (53.4%) assessments seen across the six supported hospitals is much higher than has been reported in other LMICs. Studies conducted in Kenya, Ethiopia, and Ghana have reported 17,13, and 25% improvement in HH compliance, respectively (15, 43, 44). Studies done in high income countries show varying HH compliance rates among HCWs compared to findings from our study, with some countries having a lower compliance rate while others have a higher rate (45–48). There was an improvement in HH compliance by 27 percentage points in Singapore and 31 percentage points in Russia when compared at baseline where similar interventions were implemented (48, 49). HH compliance in both baseline and endline assessment was highest in the *after touching a patient* indication—an observation consistent with other studies on HH compliance (15, 50). This phenomenon can be elucidated by the prevalence of “inherent” HH behavior, where individuals engage in HH because they personally perceive their hands as unclean. Such individuals practice hand hygiene as a means of self-protection, even beyond healthcare environments (51). In our study, the *before touching a patient* indication demonstrated the highest improvement from baseline. The same was observed in an HH implementation study conducted in Kenya (52). This may be attributed to the focused coverage on this particular indication during training and other capacity-building activities.

In our study, doctors exhibited relatively low HH compliance compared to other professionals, and the percentage improvement in mean compliance among doctors in all hospitals was lower than that achieved by nurses and laboratory professionals. That result is consistent with observations in other studies (53, 54). The largest improvement in HH compliance from baseline to endline assessment was observed among nurses. Throughout the implementation of the CQI program, it became evident that nurses showed greater engagement in the CQI initiatives compared to other professional groups, with doctors exhibiting the lowest level of participation. This variance in participation likely contributed to the noticeable differences in the demonstrated improvements in HH compliance between doctors and nurses. Other researchers have made similar observations (55).

The program prioritized *education and training* interventions on HH due to the documented benefits in enhancing HH knowledge and improving HH practices among HCWs and patients (39, 40). This can explain why *education and training* was the most improved HH multimodal strategy, which this is consistent with findings that education is the most often used method for improving HH compliance (13, 56). The next most improved multimodal strategies were *institutional safety climate* and *system change*, which was consistent with our CQI approach of establishing an enabling environment with strong hospital leadership commitment that results in the consistent promotion of HH and support for its implementation as well as the ensured availability of necessary resources (such as soap, water, and alcohol-based hand rub). Resource availability, leadership, and organizational support have been described as key elements for sustainable implementation of HH interventions (57, 58). Our study also helps address the evidence gap regarding the effectiveness of IPC interventions in Africa, as well as the lack of implementation of best practices aligned with the WHO multimodal approach, which has been previously reported (59).

### 4.3 Impact of CQI interventions on HH/IPC knowledge

Our study was able to demonstrate improvement in HH knowledge among HCWs in all hospitals. Male HCWs consistently demonstrated a relatively high HH knowledge in both baseline and endline assessments compared with female HCWs—a result that has been observed in other studies elsewhere (60). However, female HCWs demonstrated greater improvement in their HH knowledge at the endline assessment. This was expected due to the observed higher participation of female HCWs, especially female nurses, in our CQI initiatives—an observation documented by other CQI researchers (55).

The application of CQI approaches to improve HH knowledge has been described elsewhere (39, 61). In our study, no statistically significant association was seen between knowledge on HH/IPC and IPCAF and HHSAF scores. We did not identify any similar related studies that demonstrated a statistically significant relationship between HH/IPC and IPCAF and/or HHSAF in the literature. However, in a similar study on HH compliance in Indonesia, good knowledge about the HH procedure did not lead to improved HH compliance among HCWs (61). Factors related to awareness, action control, facilitation, social influence, attitude, self-efficacy, and intention might be associated with improved HH compliance (62). This finding may imply the need to interpret improvements on IPCAF and HHSAF with caution in terms of their overall impact on IPC and HH capacity and practice in health facilities.

Our findings are consistent with similar studies using the CQI approach to implement IPC and HH interventions in low-resource settings. In Brazil, the CQI approach was useful in guiding system-wide interventions for patient safety (63), and in a parallel antimicrobial stewardship program in Uganda, CQI approaches were useful in improving antimicrobial use for selected indicators in six hospitals (17).

Thus, interventions utilizing CQI techniques can be useful for helping improve HH and IPC in HCFs in resource-limited settings. This paper provides results and value in support of such an approach. The interventions were designed, implemented, and evaluated by hospital-based IPC and HH teams, demonstrating that local capacity can be built within hospital staff to institutionalize IPC and HH implementation and that the HCFs would benefit from improved IPC practices. This would then provide a basis on which IPC committees can lobby for investment in IPC programs—investment that is neglected in Uganda and many other LMICs, as evidenced by frequent stock outs of materials for implementing IPC programs (64–66). It is crucial to strengthen the capacity of available facility staff for IPC and HH implementation, with the goal of limiting the spread of resistant infections in HCFs, ensuring patient safety, containing AMR, and contributing to the quality-of-care objective of universal health coverage. Combining CQI approaches and the WHO multimodal strategy is an effective and efficient methodology for improving HH compliance in HCFs (15). The MOH can therefore adopt CQI techniques to roll out the WHO multimodal strategy for HH as one of the approaches for advancing IPC for improving patient safety and AMR containment in HCFs.

### 4.4 Study limitations

Our study was not without limitations. While it covered six hospitals, the assessments for HCW knowledge on HH and IPC sampled 20 HCWs from each facility. We recognize that this is a small sample compared to other studies. We suggest that future studies should be broader, covering more hospitals and more HCWs assuming more resources and funding are made available. Moreover, the healthcare workers assessed their respective facilities and that might introduce biases, as some responses provided by the HCFs cannot be easily verified. Cross-validation processes can mitigate such limitations, but we cannot completely eliminate them. We therefore suggest that future assessments should be verified by external assessors.

While we prioritized departmental/unit heads and champions for IPC/HH committees and teams memberships in the HCFs, the seniority of these heads often meant they were too busy to fully engage in the intervention efforts. In contrast, the champions were the primary drivers of the intervention. Attempts to update the membership by replacing these departmental/unit heads proved too difficult, leading to setbacks in some intervention activities. Therefore, we recommend prioritizing champions, who are motivated and actively engaged, for inclusion on IPC/HH committees and teams, while ensuring they operate under the supervision of senior staff to drive improvement actions effectively.

Additionally, it is important to note that the HH compliance assessments did not fully consider the Hawthorne effect, wherein behavior may change due to awareness of being observed. In this case, HCWs were aware of being observed, as per the WHO methodology, which may have influenced their HH compliance to a certain degree (67). Furthermore, we suggest that additional parameters—e.g., ward infrastructure, perception of HCWs’ and hospital senior managers’ attitudes toward IPC, and other ongoing interventions—be included while doing this type of study. This would provide a more holistic status of IPC and HH in HCFs.

Finally, we opted not to include assessments of HAI as outcome indicators, primarily because of ongoing discussions within the country regarding HAI tools and assessment methodologies. However, future implementation studies should incorporate HAI indicators when executing CQI plans for IPC and HH.

## 5 Conclusion

The implementation of the WHO IPC and the HH improvement strategy using a CQI approach led to significant enhancements in IPC, HCW compliance and knowledge and HH capacity and practices across the six hospitals in resource-limited settings. While there were slight variations among hospitals and between IPC and HH capacities and practices, the effectiveness of hospital-led interventions was evident. These improvements were largely driven by champions (particularly nurses), the adaptation of standardized tools and approaches, and the government’s response to the COVID-19 pandemic at the healthcare facilities. Targeted interventions resulted in notable improvements in specific core components, multimodal strategies, and HH indications. However, further long-term studies are needed to explore the factors necessary for sustaining IPC and HH improvement.

## Data Availability

The raw data supporting the conclusions of this article will be made available by the authors, without undue reservation.
